# SARS-CoV-2 in animals: what about the cat?

**DOI:** 10.1080/01652176.2021.1958393

**Published:** 2021-08-03

**Authors:** Ana Izabel Passarella Teixeira

**Affiliations:** Tropical Medicine Center, University of Brasília, Brasília, Brazil

The characteristics of the origin of severe acute respiratory syndrome coronavirus 2 (SARS-CoV-2) has puzzled researchers, especially in regards to its zoonotic potential. This paucity of information has driven me to pursue scientific discoveries on this topic. After reading the paper “SARS-CoV-2 in animals: potential for unknown reservoir hosts and public health implications” by Dr. Sharun et al^1^, I was very pleased with their results, and I would like to share some thoughts about their findings. The authors correctly pointed out that, besides mustelids, felids should also be considered as a matter of concern in terms of animal and public health. The COVID19 virus infects and multiplies within animal hosts; there is thus a need for surveillance to track virus prevalence and circulation and the possibility of a spill-back to humans.

The felid in close contact with humans is the domestic cat, and it has thus been the subject of several studies. However, the scientific evidence from these studies is conflicting and paradoxical. Some studies have shown that cats can be infected with SARS-CoV-2 and produce specific antibodies against the viral antigens. Cats can be infected by their owners, and cat-to-cat transmission has been demonstrated experimentally (Garigliany et al. 2020; Halfman et al. 2020; Sailleau et al. 2020; Shi et al. 2020). In experimental infections, most cats remain asymptomatic, even with high infective doses (Halfman et al. 2020; Shi et al. 2020). When symptoms develop, they are the general symptoms of viral infection. The most serious symptoms are similar to those of human cases of SARS-CoV-2. For example, in one report of a cat that lived with a human with COVID-19, the cat developed cough, difficulty breathing, and prostration, which evolved with spontaneous cure (Garigliany et al. 2020; Segalés et al. 2020). Another example is that of a cat in Italy with several comorbidities that lived with a human with COVID-19, who developed the ground-glass pattern in pulmonary images (Musso et al. 2020). Klaus et al. (2021) further reported the case of a cat with an intestinal lymphoma (a comorbidity) with symptomatic COVID-19, presenting with sneezing, coughing, and ocular discharge. In addition, Villanueva-Saz et al. (2021), duly quoted by Dr. Sharun, that SARS-CoV-2 cats were seropositive for another infectious disease that can cause immunosuppression. Therefore, the cases of COVID-19 that occur in cats are similar to those in humans.

In surveys or other kinds of studies aiming to detect antibodies against SARS-CoV-2 or antigens and to explore a possible epidemiological role in the epidemiological chain of transmission of this virus, there have been conflicting findings. In some studies, there was low prevalence or no detection. They inferred that the epidemiological role of cats should be none or rather limited (Temmam et al. 2020; Deng et al. 2020). However, other studies have shown that it must play a noteworthy epidemiological role. A higher seroprevalence was observed in cats during the second COVID-19 wave than during the first wave. The number of human cases was also higher in the second wave (Michelitsch et al. 2021). Cats with infected owners were shown to have a low prevalence of SARS-CoV-2 antibodies, while stray cats had none (Spada et al. 2021). Alternatively, case reports of symptomatic cat cases, such as that of Musso et al. (2020), were unable to establish how the cat was infected, as the cat was able to go outdoors. A report of cats with neutralizing antibodies living with owners with COVID-19 has also been published (Jara et al. 2021).

Thus far, there has been no definitive evidence of cat-to-human transmission. However, as explored above, there is evidence of human-to-cat transmission, and also mink-to-cat transmission and mink-to-human transmission (Oude-Munninck et al. 2021; Van Aart et al. 2021). However, it would be difficult to detect this evidence. Totton et al. (2021) stated that to demonstrate cat-to-human transmission, the following conditions should be verified: 1. An effective quarantine period, followed by negative PCR and serologic testing, to eliminate the potential for undetected infection in the person, and 2. The person must remain isolated from all other sources of SARSCoV-2 from the start of the effective quarantine period, throughout the exposure to the infected cat, development of symptoms, and diagnosis.

After my research I was left with one question: considering all these findings, is it reasonable to think that cat-to-human transmission could occur? I think it can occur, but as the evidence points out, it should be in a low rate. However, if (and it’s a big if) it does occur, although at a low rate, when we think of the entire population of cats, it may have an epidemiological relevance.

All these findings and questions highlight the importance of the One Health Approach in matters of public health. The emergence of SARS-CoV-2 and other pandemic viruses with zoonotic characteristics has already shown us how deeply human and animal health are interconnected (Dhama et al. 2020). I believe that by further exploring this health concept, we will be able to develop tools to control this pandemic and prevent others in the future.


Ana Izabel Passarella Teixeira *Tropical Medicine Center, University of Brasília, Campus Universitário Darcy Ribeiro, Brasília, Federal District, Brazil* ana.teixeira@unb.br


## Note

1 Veterinary Quarterly, 2021, vol. 41, N. 1, 181–201 https://doi.org/10.1080/01652176.2021.1921311
